# Special Issue: “Functional Dendrimers”

**DOI:** 10.3390/molecules21081035

**Published:** 2016-08-09

**Authors:** Donald A. Tomalia

**Affiliations:** 1 Department of Chemistry, University of Pennsylvania, Philadelphia, PA 19104, USA; 2 Department of Physics, Virginia Commonwealth University, Richmond, VA 23284, USA; 3 National Dendrimer & Nanotechnology Center, NanoSynthons LLC, 1200 N. Fancher Avenue, Mt. Pleasant, MI 48858, USA; donald.tomalia@gmail.com; Tel.: +1-989-317-3737

**Keywords:** dendrimers/dendrons, hyperbranched polymers, major polymer architectures, dendritic effects, critical nanoscale design parameters (CNDPs), nanoperiodic property patterns, new emerging dendrimer properties

## Abstract

This special issue entitled “Functional Dendrimers” focuses on the manipulation of at least six “critical nanoscale design parameters” (CNDPs) of dendrimers including: size, shape, surface chemistry, flexibility/rigidity, architecture and elemental composition. These CNDPs collectively define properties of all “functional dendrimers”. This special issue contains many interesting examples describing the manipulation of certain dendrimer CNDPs to create new emerging properties and, in some cases, predictive nanoperiodic property patterns (i.e., dendritic effects). The systematic engineering of CNDPs provides a valuable strategy for optimizing functional dendrimer properties for use in specific applications.

Dendritic polymers, recognized as the fourth major architectural class of polymers after the three well-known traditional types (i.e., I: linear, II: cross-linked and III: branched polymers), have grown dramatically over their 27-year history. To date, over 50,000 patent and literature citations related to this important class of dendritic polymers have appeared [1].

The first theoretical concept for random hyperbranched, dendritic polymers (i.e., tree-like architecture) was advanced in the 1950s by P. Flory [2]; however, the actual synthesis of Flory-type structures did not occur until the late 1980s [3,4]. The history of more “structure-controlled” dendritic polymers (i.e., dendrons/dendrimers) began with the synthesis of low molecular weight dendrimer precursors (i.e., cascade molecules) in 1978 [5], followed by the first divergent synthesis of complete structure-controlled, macromolecular (i.e., >1 nm or >1 kd) dendrimer families in 1984–1985 [6,7] and the first convergent dendrimer synthesis in 1990 [8]. Subsequently, the general category of dendritic polymers was expanded to include at least four dendritic subcategories generally characterized by their discrete nanoscale dimensions and varying degrees of dispersity and structural control. In ascending order of structural control, they include: (1) random hyperbranched polymers; (2) dendrigrafts; (3) dendrons and (4) dendrimers.

Structurally controlled dendritic polymers (i.e., sub-categories 2–4) possess at least six well-defined nanoscale features referred to as *critical nanoscale design parameters* (CNDPs) [9,10,11,12,13]. These CNDPs include six discrete features: *sizes, shapes, surface chemistries, flexibilities/rigidities, architectures and elemental compositions*. These CNDPs collectively define critical “functional features” which are unique characteristic of all dendrimers. More importantly, it is now known that these CNDPs may be systematically manipulated to produce an array of new emerging properties and predictable *nanoperiodic property patterns* (i.e., *dendritic effects)* [11] that may be desirable/critical for many important commercial applications [1], as well as nanomedicine [14,15,16].

This current *Molecules* Special Issue*,* organized by Prof. Dr. Ashok Kakkar (McGill University), focuses on *Functional Dendrimers* and, as such, the engineering of dendrimer CNDPs. Within the context of this “functional dendrimer” criteria, Kakkar has organized an overview of 10 excellent articles/reviews, authored by many recognized pioneers and active researchers in the dendrimer field, wherein they have investigated or manipulated dendrimer CNDPs to produce a wide range of new properties or nanoperiodic property patterns.

For example, the importance of polymeric architecture is demonstrated, wherein various dendritic structures were analyzed mathematically as a function of their topological indices (i.e., Randic, atom-bond connectivities, etc.) in an effort to determine quantitative nanoscale structure activity/property relationships (QNSAR) and predictable activity patterns based on hyperbranched architecture [17].

The effects of polymeric architecture types (i.e., linear, hyperbranched, dendrimeric) as well as macromolecular sizes on various properties (i.e., solubilities, viscosities, thermal properties) were reported by A. Morikawa et al. [18].

The effects of size, interior composition and perhaps flexibility/rigidity or architecture associated with in vitro interactions of two different dendrimer families (i.e., PAMAM versus triazine dendrimers) on human platelets were reported by E. Simanek et al. [19].

The influence of the triazine dendrimer (G = 5) surface chemistry on coacervation and metal encapsulation properties was examined by E. Simanek et al. [20], wherein they noted that hydrophobic surface moieties dramatically influenced liquid-liquid phase transitions associated with lower critical solution temperatures (LCST).

A review by R. Sanyal, A. Sanyal et al. [21] describes the critical roles that architecture and surface chemistry play when using dendrons and dendrimers as discrete building blocks in the construction of functional hydrogels for applications such as sensing, drug delivery and tissue engineering.

The role of dendritic architecture, size, as well as surface chemistry/linkers for presenting various prodrugs to improve pharmacokinetic properties is described in an extensive review by E. I. Ferreira, J. Giarolla et al. [22]. Improved targeting of the disease site, controlled release and decreased toxicity may be obtained by properly engineering certain dendrimer CNDPs for pharmaceutical applications.

An extensive review article by A.-M. Caminade and J.-P. Majoral [23] describes the unique features that result from engineering bifunctional dendrimer and dendron domains (i.e., Janus-type architectures) possessing phosphorous-functionalized interiors and surface chemistries. In many cases, optimized structures and properties may be obtained for use in catalysis, fluorescence and biological applications.

An article by M. Malkoch et al. [24] describes the use of synthetic green chemistry protocols involving protect/deprotect, click and novel fluoride-assisted esterification chemistry (FPE), wherein either biotin or azido moieties could be readily introduced at the focal point or on the dendritic surface in high yield to produce monodispersed bis-MPA dendrons using less toxic solvents.

A highly versatile triazine dendrimer was CNDP-engineered and synthesized to contain at least four maleimide surface groups available for a variety of bioconjugation possibilities (i.e., drugs or targeting moieties), and a core moiety (i.e., DOTA) suitable for MRI imaging was reported by E. Simanek et al. [25]. These uniquely functionalized dendrimers are expected to play a role in the design and construction of potentially important theranostic devices.

R. Roy et al. [26] report on an investigation describing the synthesis of a very innovative new class of dendritic polyols derived from high-multiplicity core moieties (i.e., pentapropargylated β-d-glucopyranoside, using an A_5_-type monomer reactant) in various click reactions with 2-azidoethyl tetra-*O*-allyl-β-d-glucopyranoside as an AB_4_-repeating moiety. These very dense, highly congested dendritic structures were further modified using thiol-ene and thiol-yne click reactions with alcohols to produce the final dendritic polyols. Molecular dynamic simulation supported the assumption that the resulting polyols possess a dense structure.

In conclusion, this *Molecules* Special Issue presents a rich collection of recent advances that clearly demonstrates the evolving tapestry of possible new, emerging, functional dendritic polymer properties and nanoperiodic property patterns that may be obtained by simply modifying six *critical nanoscale design parameters* (CNDPs) associated with dendrimers.
